# Effect of Oxygen Partial Pressure on the Phase Stability of Copper–Iron Delafossites at Elevated Temperatures

**DOI:** 10.3390/ma11101888

**Published:** 2018-10-02

**Authors:** Thomas Stöcker, Ralf Moos

**Affiliations:** Department of Functional Materials, Zentrum für Energietechnik (ZET), University of Bayreuth, 95440 Bayreuth, Germany

**Keywords:** delafossite, Ellingham diagram, phase stability, high temperature thermoelectric materials, thermoelectric generator (TEG)

## Abstract

Oxide-based materials are promising candidates for use in high temperature thermoelectric generators. While their thermoelectric performance is inferior to commonly used thermoelectrics, oxides are environmentally friendly and cost-effective. In this study, Cu-based delafossites (CuFeO_2_), a material class with promising thermoelectric properties at high temperatures, were investigated. This work focuses on the phase stability of CuFeO_2_ with respect to the temperature and the oxygen partial pressure. For this reason, classical material characterization methods, such as scanning electron microscopy, energy dispersive X-ray spectroscopy, and X-ray diffraction, were combined in order to elucidate the phase composition of delafossites at 900 °C at various oxygen partial pressures. The experimentally obtained results are supported by the theoretical calculation of the Ellingham diagram of the copper–oxygen system. In addition, hot-stage X-ray diffraction and long-term annealing tests of CuFeO_2_ were performed in order to obtain a holistic review of the phase stability of delafossites at high temperatures and varying oxygen partial pressure. The results support the thermoelectric measurements in previous publications and provide a process window for the use of CuFeO_2_ in thermoelectric generators.

## 1. Introduction

In past years, huge efforts have been undertaken to cope with global warming and climate change, mainly originating of ever-growing economics and societies in industrial countries. By doing so, it is a significant challenge to recycle the waste-heat, accountable for up to 60% of all energy losses, using efficient energy converters. Thermoelectric generators (TEG) make use of the Seebeck effect to directly convert thermal into electrical energy. Various material combinations have been investigated in the last decades, especially for applications in mid-temperature ranges [1,2,3,4,5,6,7]. Thereby, various material combinations have been used to increase conversion efficiency, but not focusing on cost efficiency, availability, and sustainability.

Hence, oxide thermoelectrics have attracted much attention in recent years for use in thermoelectric generators [8,9,10,11,12,13,14,15,16,17,18]. Whereas commonly used material classes such as chalcogenides [19,20,21,22,23,24,25,26,27,28,29], skudderudites [30,31,32], and polymers [33,34,35,36,37] exhibit good thermoelectric performance at low- and mid-temperature ranges, oxides show their advantages at elevated temperatures above 700 °C. Above all, oxide materials follow the prevailing trend to substitute costly and less abundant thermoelectrics in favor of inexpensive materials. Whereas their thermoelectric performance might be inferior to prevalently used materials, oxides exhibit a remarkable relationship between thermoelectric performance and cost. Thus, thermoelectric oxides make use of their inherent advantages where no high-performance thermoelectric efficiency is required, but the application of cost-effective and environmentally friendly materials are a must.

Lately, several promising groups of oxide thermoelectrics were reported to have a considerable good thermoelectric performance. Among those, cobaltites such as NaCo_2_O_4_ [38,39] or Ca_3_Co_4_O_9_ [40,41,42,43,44,45,46,47,48] were seen as potential *p*-type material in thermoelectric generators. However, they are not stable against temperature cycling and require complex synthesis routes. Concerning *n*-type thermoelectrics, especially titanates such as SrTiO_3_, show the most promising thermoelectric properties. While their mobility is comparatively low, the effective mass is notably high [49,50,51,52], resulting in a very good thermoelectric efficiency. Further improvement was possible by using natural superlattices of SrTiO_3_, the so-called Ruddlesden–Popper phases [53,54,55]. Current research also focuses on layered In_2_O_3_ composites, enabling sustainable, cost-effective, and efficient *n*-type thermoelectrics for thermoelectric generators. Korotenchok et al. provide a highly topical review on oxide thermoelectrics with focus on the above-mentioned In_2_O_3_ [56].

Recently, some studies describe copper–iron oxides and claim them as promising thermoelectric materials due to their high Seebeck coefficient, while sustaining a high electrical conductivity and thermal stability [57,58,59,60]. Former studies have focused on the thermoelectric performance and the electrical conductivity of the delafossite-type oxide CuFeO_2_, its dependence on the oxygen partial pressure at high temperatures, and on the novel aerosol deposition coating technique [61,62,63,64]. In this work, we focus on the phase stability of delafossites at elevated temperatures and under varying oxygen concentrations. Whereas Stöcker et al. showed an in-situ phase transition by measuring the thermopower of CuFeO_2_ with increasing oxygen partial pressure, this study aims on a holistic material characterization of CuFeO_2_ and its stability for their application in thermoelectric generators at high temperatures.

## 2. Materials and Methods

Delafossite powders were prepared in a conventional mixed-oxide technique. In order to obtain a high purity starting material, a synthesis route as reported in [64] was chosen, describing the formation of CuFeO_2_ with no impurities or secondary phases. As starting materials, copper(I) oxide (99.9%, Alfa-Aesar, Karlsruhe, Germany) and iron(III) oxide (99%, Alfa-Aesar, Karlsruhe, Germany) were used and processed in a wet planetary ball mill (Fritsch, Idar-Oberstein, Germany) with cyclohexane as solvent. The stoichiometric mixtures were ball-milled for 4 h in order to homogenize the materials. After removing the solvent in a rotary evaporator (Heidolph Instruments, Schwabach, Germany), the powders were calcined in a high-temperature furnace (STF/15 450, Carbolite-Gero, Germany) at 1050 °C for 12 h in a mixed gas atmosphere of 0% O_2_, 1% O_2_, and 10% O_2_ in nitrogen. The obtained delafossite powders were again reground in a planetary mill, sieved with a 90 µm screen in order to reduce agglomerates, and dried in a furnace at 200 °C. The phase composition of the obtained powders was elucidated by using an X-ray diffraction system (PANalytical, Almelo, The Netherlands) operating with CuK_α_ radiation (1.541874 Å) within 2*θ* = 25° … 60° at a step size of 0.02°. 

In order to evaluate the phase composition of CuFeO_2_ as a function of *p*O_2_ at elevated temperatures, CuFeO_2_ brick shaped pellets were cold-pressed uniaxially [64] and annealed at 900 °C under different oxygen concentrations (0, 1, 5, 10, 20, and 100%) mixed in nitrogen for 12 h. While the oxygen diffusivity of CuFeO_2_ is low, previous investigations have shown that an annealing time of 12 h is sufficient for the samples to reach an equilibrium [64]. A scanning electron microscope (LEO 1450 VP, Zeiss, Oberkochen, Germany) was used for energy dispersive X-ray spectroscopy (EDX) and back-scattered electron (BSE) imaging of the samples. Additionally, pellets were reground for X-ray diffraction analysis. This combination of XRD and EDX/BSE studies facilitates an in-depth analysis of the *p*O_2_ influence on the crystal structure and phase composition of CuFeO_2_. Additionally, the results were combined with theoretical calculations of the predominance diagram for the copper–iron–oxygen system in order to verify the experimental findings. 

Since delafossites are possible candidates for high-temperature thermoelectrics, hot-stage XRD (D8 ADVANCE, Bruker with hot stage HTK 1200-N, Anton Paar, CuK_α_ radiation) analysis of CuFeO_2_ powder calcined at 1% O_2_ in nitrogen was conducted from 20 to 900 °C under nitrogen gas atmosphere. After an equilibration time of 30 min, XRD patterns were recorded at discrete temperature levels, so possible phase changes could be investigated in operando. Finally, long-term tests of CuFeO_2_ were performed, by thermally treating bulk samples that were free from secondary phases for 96 h in nitrogen. These specimens were investigated by a combination of XRD and SEM analysis.

## 3. Results and Discussion

**Figure 1:**
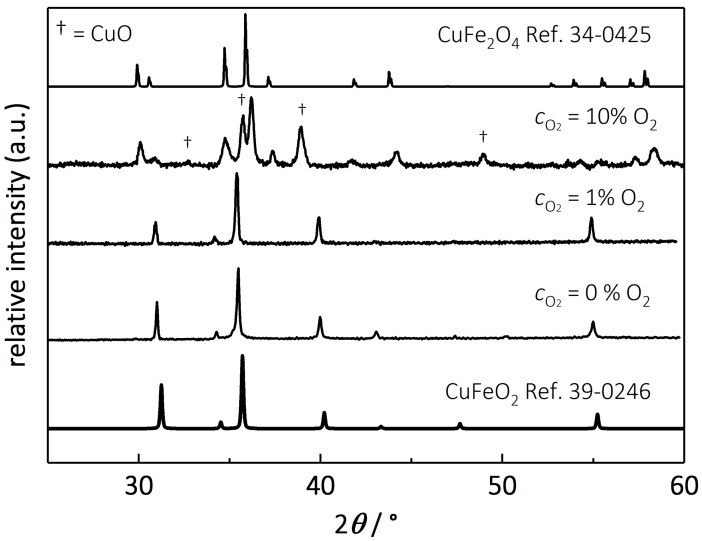
XRD patterns of CuFeO_2_ calcined under nitrogen as well as 1% and 10% oxygen mixed in nitrogen. Additionally, the reference spectra of CuFeO_2_ (JCPDS 39-0246) and CuFe_2_O_4_ (JCPDS 34-0425) are depicted.

Figure 1 shows the XRD patterns of the calcined delafossite powders and the reference spectrum of delafossite CuFeO_2_ (JCPDS 39-0246) and indicates no impurities i.e., no secondary phases for samples processed with 1% O_2_ mixed in nitrogen. This result is in good agreement with previous investigations [64,65,66,67], whereas the calcination in pure N_2_ leads to an elemental copper phase, due to the reduced oxygen partial pressure. Here, we focus on the influence of an elevated oxygen partial pressure on the phase stability of delafossites. In contrast to characteristic diffraction reflexes of delafossite, CuFe_2_O_4_, and possibly CuO (marked as †) are predominant for samples annealed under 10% oxygen mixed with nitrogen. 

**Figure 2:**
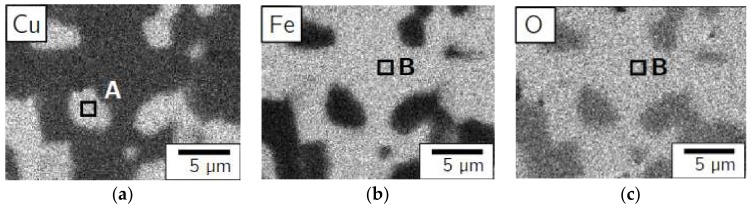
EDX pattern of the delafossite, annealed at 900 °C for 12 h in a gas mixture of 10% oxygen in nitrogen. (**a**) copper; (**b**) iron; (**c**) oxygen.

In order to verify the phase transition of CuFeO_2_ to CuFe_2_O_4_ and CuO, energy dispersive X-ray spectroscopy investigations were conducted on samples of CuFeO_2_ calcined in 1% oxygen and annealed in different oxygen atmospheres. The EDX mapping of characteristic regions of a polished cross-sectional sample, annealed at 900 °C for 12 h in a gas mixture of 10% oxygen in nitrogen, is depicted in Figure 2. It indicates copper, iron, and oxygen. In the element distribution images, two homogeneous distributed phases can be seen, whereas the brightness in the images represents qualitatively the concentration of the corresponding element.

The first phase exhibits no iron and is depicted in Figure 2a as white zones, consisting of copper and oxygen. Contrarily, the second phase solely contains iron and oxygen, illustrated in Figure 2b,c. In order to elucidate the phase composition, quantitative EDX analyses for the cuprous phase (marked as A in Figure 2a) and the ferrous phase (marked as B in Figure 2b,c) were conducted. The element mole fraction for the two regions and the theoretical compositions of CuO and CuFe_2_O_4_ are listed in Table 1. While the deviation of oxygen in EDX spectroscopy can be up to 3 mol %, the results provide a rough estimation of the phase compositions.

The findings of the EDX analysis are in line with the results of the XRD recordings. The delafossite is subject to a *p*O_2_-dependent phase transition at elevated temperatures to CuO and CuFe_2_O_4_. Our findings reveal a fully completed conversion when annealing CuFeO_2_ in a gas mixture of 10% oxygen in nitrogen. Against the background of thermoelectric performance of delafossites in thermoelectric generators, this phase change limits the usage of CuFeO_2_ in oxygen-rich atmospheres. While the conduction mechanism of the delafossites is *p*-type, the arising mixed phase of CuO/CuFe_2_O_4_ shows a mixed conduction, since copper (I) oxide is an *n*-type and the cuprospinel CuFe_2_O_4_ is a *p*-type semiconductor, resulting in bipolar thermoelectric effects and therefore reducing the overall thermoelectric performance [68,69]. Previous defect-chemical studies with variations in the oxygen partial pressure on thin delafossite films prepared by aerosol deposition also showed an abrupt change in the conductivity at characteristic *p*O_2_ levels at high temperatures, and this change results in bipolar thermoelectric effects [64]. These findings are supported by the results in the present work. They provide a process window for the usage of CuFeO_2_ as a thermoelectric material at elevated temperatures. 

**Figure 3:**
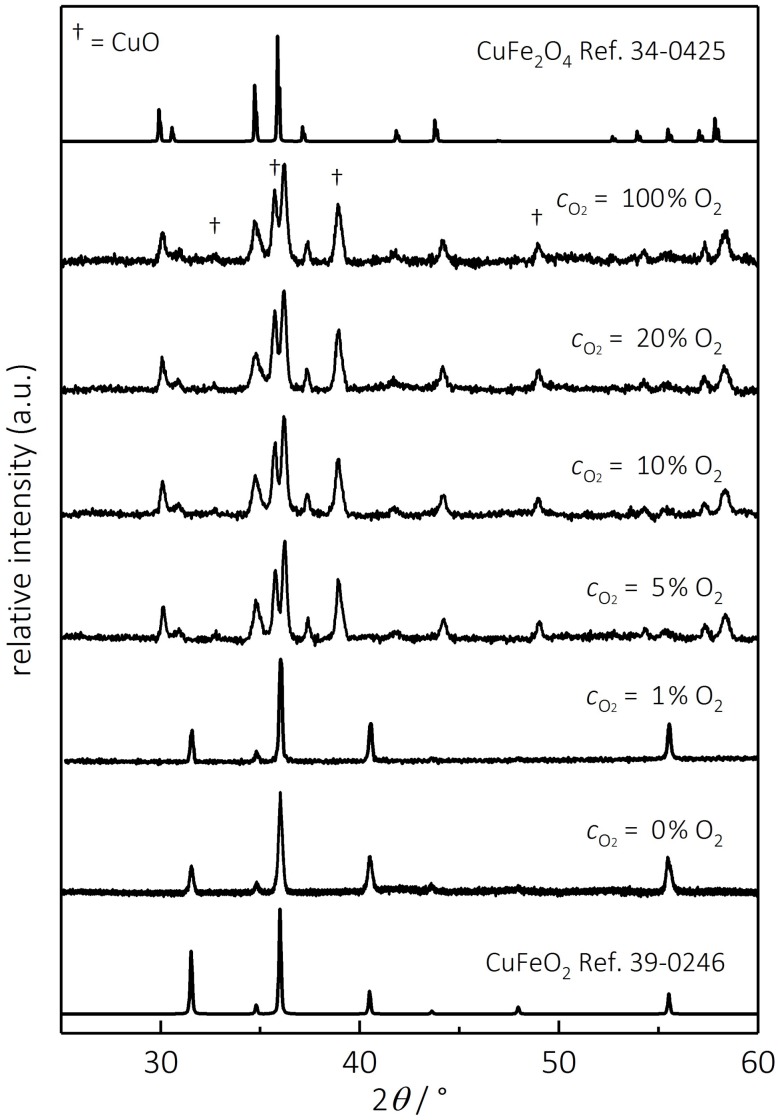
XRD patterns of CuFeO_2_ annealed in 900 °C for 12 h under different oxygen–nitrogen gas mixtures. Additionally, the reference spectra of CuFeO_2_ (JCPDS = 39-0246) and CuFe_2_O_4_ (JCPDS = 34-0425) are displayed.

To specify the phase stability threshold for delafossites, XRD patterns of CuFeO_2_ samples annealed at 900 °C in graduated oxygen–nitrogen gas atmospheres were taken. Figure 3 shows the resulting diffraction patterns and the reference spectra of CuFeO_2_ (JCPDS 39-0246) and CuFe_2_O_4_ (JCPDS 34-0425).

Delafossits annealed in 0% and 1% oxygen exhibit no secondary phases, while all samples starting from an oxygen concentration of 5% show a phase transition to CuFe_2_O_4_ and CuO, with no additional phase changes at higher oxygen partial pressures and no remaining CuFeO_2_. These findings correspond well with defect-chemical characterizations of CuFeO_2_, where the phase transition at 900 °C was observed at oxygen concentrations between 1 and 3.1% [64]. Hence, these results provide a rough process window in the context of oxygen stability for delafossites at 900 °C. In order to refine these experimental findings, calculations of the equilibrium equation for the oxidization of CuFeO_2_ according to Equation (1) lead to a theoretical stability window for delafossites. 

4 CuFeO_2_ + O_2_ ↔ 2 CuFe_2_O_4_ + 2 CuO. (1)

Equation (1) describes the observed phase transition under oxidizing atmospheres. The Gibbs energy Δ*G* of a system is determined by the following expression [70]:

Δ*G* = Δ*G*^0^ + *RT* ln(*K*_eq_). (2)

Δ*G*^0^ stands for the Gibbs free energy change per mole of reaction for unmixed reactants and products at standard conditions, *R* for the gas constant, *T* for the absolute temperature, and *K*_eq_ denotes the equilibrium constant. If the system is in chemical equilibrium, the Gibbs free energy of Reaction (1) can be calculated as follows, assuming solid solutions for the reactants [71]:

Δ*G*^0^ = −*RT* ln(*p*O_2_). (3)

The Gibbs free energy per mole of formation, Δ*G*^0,1^ for Reaction (1), can be described as [71]

Δ*G*^0,1^ = −22115 J mol^−1^ + 160.20 J mol^−1^*K*^−1^·*T*. (4)

**Figure 4:**
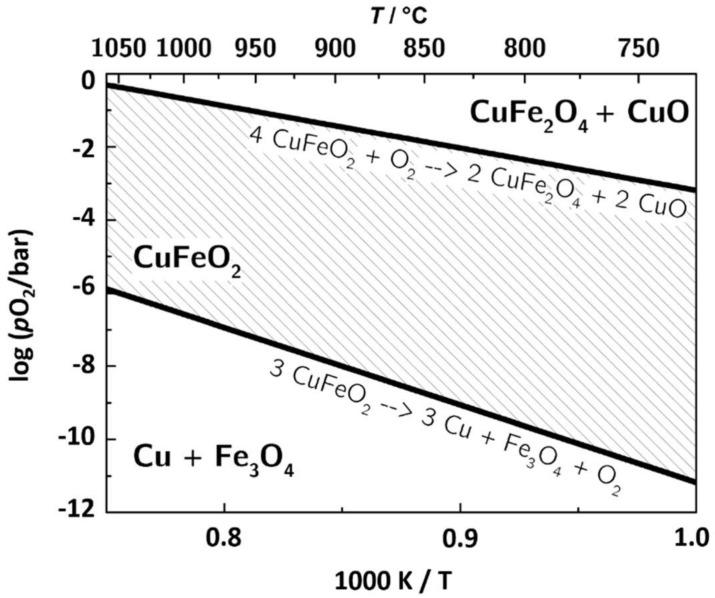
Ellingham diagram of the CuFeO_2_, CuFe_2_O_4_, CuO, and the Fe_3_O_4_ system, respectively.

Combined with Expression (3), this yields the Ellingham diagram for Reaction (1) shown in Figure 4.

In addition to the oxidation reaction, the equilibrium curve for the reduction of CuFeO_2_ at low *p*O_2_ corresponding to Expressions (5) and (6) is displayed [71]. The expressions are as follows:

3 CuFeO_2_ ↔ 3 Cu + Fe_3_O_4_ + O_2_ (5)

Δ*G*^0,2^ = −405350 J mol^−1^ + 191.40 J mol^−1^*K*^−1^·*T*. (6)

The calculated Ellingham diagram is in good agreement with the experimental findings in this work, verifying the observed phase transitions by XRD and EDX with theoretical calculations. It also supports the thermoelectric measurements in the previous publications [64,72]. For 700, 800, and 900 °C, Table 2 lists upper and lower limit of *p*O_2_ for the stability of CuFeO_2_. At higher oxygen concentrations, the delafossite oxidizes to CuFe_2_O_4_ and CuO and at low *p*O_2_, it decomposes to Cu, Fe_3_O_4_, and O_2_. While the reduction of CuFeO_2_ can be observed during the synthesis at 1050 °C under nitrogen [64], the employed nitrogen (N_2_, 5.0) contains up to 3 ppm oxygen (*p*O_2_ = 3 × 10^−6^ bar), resulting in no decomposition at 900 °C.

These results yield a stability region for the delafossite, depicted as a hatched area in Figure 4, as a function of temperature and oxygen partial pressure. For example, at temperatures above 700 °C, CuFeO_2_ is not stable in ambient air, so an encapsulation would be required if delafossites are used as thermoelectric materials in high-temperature thermoelectric generators.

**Figure 5:**
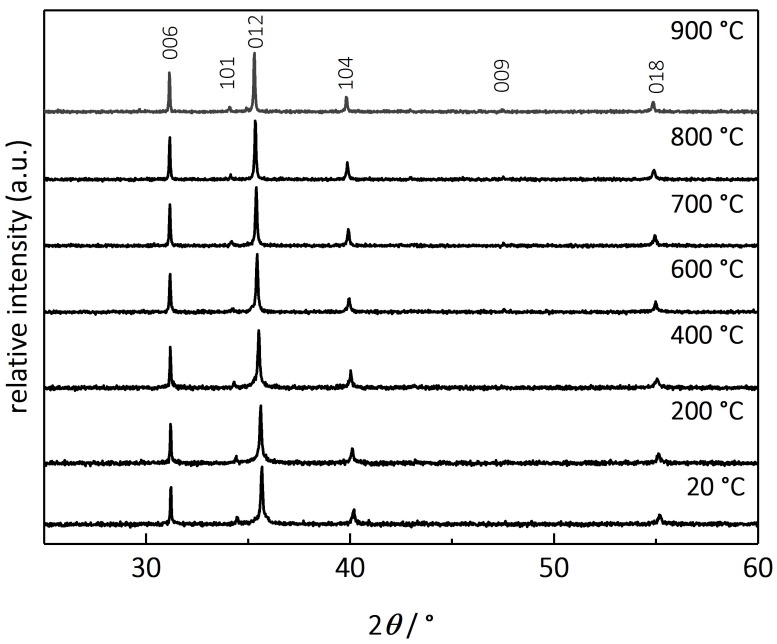
High-temperature X-ray diffraction patterns of CuFeO_2_, measured in nitrogen at discrete temperature levels as indicated.

In order to verify the long-term stability of CuFeO_2_ and to rule out occurring secondary phases when heating up the material, high-temperature XRD was conducted on delafossites in a nitrogen gas atmosphere. Figure 5 depicts the diffraction pattern at different temperatures. Owing to grain growth, the reflexes, respectively the FWHM, broaden with increasing temperature, but no impurities or secondary phases can be detected, confirming that no phase transformations occur from room temperature to 900 °C.

**Figure 6:**
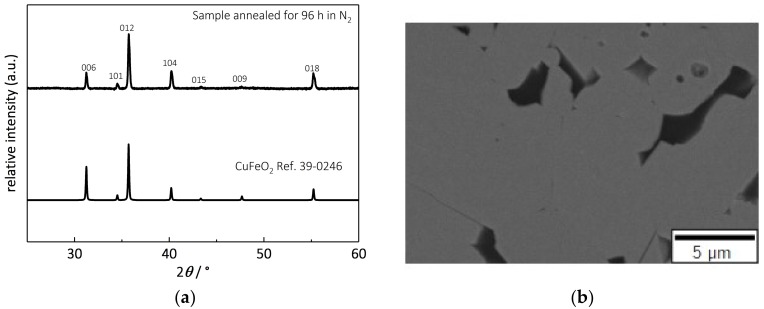
Delafossite annealed for 96 h at 900 °C in nitrogen. (**a**) Diffraction and reference patterns of CuFeO_2_ for comparison; (**b**) scanning microscope image (BSE detector) of the annealed sample showing no secondary phases. The dark areas are voids resulting from sample preparation.

The previous investigations were conducted on samples that were annealed for 12 h in the corresponding gas atmospheres. Especially at high temperatures, aging effects and the interdependency of the substrate with the delafossites, caused by ion exchanges [73,74], may deteriorate the material leading to poorer thermoelectric properties. For this reason, long-term tests of CuFeO_2_ with no secondary phases on alumina substrates were performed. Figure 6a shows the diffraction pattern of a delafossite annealed in nitrogen at 900 °C for 96 h, revealing neither impurities nor any phase changes. This result is confirmed by the BSE image in Figure 6b, where solely CuFeO_2_ was observed. Neither elemental copper, nor copper–alumina-spinel phases, resulting from possible interactions between the delafossite and the alumina substrate, were detected. The black areas are voids resulting from sample preparation.

## 4. Conclusions

In the present study, the phase stability of delafossites at high temperatures and elevated oxygen concentrations was studied. Previous investigations indicated a change in the conduction mechanism of CuFeO_2_ at high oxygen concentrations, assuming a phase change of the material and reducing the thermoelectric performance. In this work, classical material characterization methods were employed to elucidate this effect in detail. The experimentally obtained results indicated the proposed transition of CuFeO_2_ to CuFe_2_O_4_ and CuO being caused by a further oxidation of the delafossites with increasing oxygen partial pressure. Both XRD and SEM/EDX analysis confirmed this reaction, thus limiting the usage of CuFeO_2_ as high-temperature thermoelectric material to a small process window with respect to oxygen concentration of the ambience and temperature.

While the experimental data gave a rough estimation of the boundaries of the stability of delafossites, theoretical calculations of the Ellingham diagram for the CuFeO_2_, the CuFe_2_O_4_, and the CuO system, respectively, lead to a detailed *p*O_2_ range in which the delafossites can be considered phase-stable. At temperatures above 700 °C, the upper *p*O_2_ limit for the phase change is lower than the oxygen partial pressure of ambient air. Hence, the usage of delafossites as thermoelectric material at high temperatures is limited to low oxygen environments or requires the thermoelectric generator to be encapsulated. 

Further investigations and thermoelectric characterizations on the occurring CuFe_2_O_4_/CuO bipolar phase may lead to an eligible *n*-type counterpart for delafossites in thermoelectric generators, since both thermoelectric materials could be processed from the same raw material. For that matter, both thermoelectric legs of a thermoelectric generator can be assembled from CuFeO_2_ as starting material. While the *p*-type legs are protected from an oxidizing gas atmosphere, thus remaining delafossites, the *n*-type legs undergo a phase transition as described in this work, leading to a thermoelectric generator of *p*-type CuFeO_2_ and *n*-type CuFe_2_O_4_/CuO. 

## Figures and Tables

**Table 1:** Calculated element mole fraction based on the quantitative EDX analysis of the two regions marked in Figure 2 and the theoretical composition of CuO and CuFe_2_O_4_.

Element	Region A	Region B	CuO	CuFe_2_O_4_
Copper	49 mol %	15 mol %	50 mol %	14 mol %
Iron	-	24 mol %	-	29 mol %
Oxygen	51 mol %	61 mol %	50 mol %	57 mol %

**Table 2:** Stability limit of CuFeO_2_ for three characteristic temperatures, corresponding to the Ellingham diagram shown in Figure 4. The upper (oxidization) and lower (reduction) limits are listed.

Temperature	700 °C	800 °C	900 °C
Oxidation/log(*p*O_2_/bar)	−3.50	−2.40	−1.48
Reduction/log(*p*O_2_/bar)	−11.76	−9.74	−6.00
